# 

**Published:** 1869-07

**Authors:** 


### EDITORIAL DEPARTMENT.

* The Ninth Annual Meeting of the American Dental Association, will be held at Saratoga Springs, N. Y., commencing Tuesday August 2nd 1869, at ten o'clock A. M.

The following form of certificate was adopted at the last meeting of the Assocication.

This certifies that ——— was duly appointed a delegate to the American Dental Association on —— day of —— and that said ———is a Dentist of good character and standing, and at this time in regular practice.

No delegate will be admitted without he answers the requirements of this certificate, which he must bring with him, and present in person.

Accommodations will be secured for those giving early notice to Dr. J. G. Ambler, No. 25 West Twenty-third St., N. Y. City.

There is reason to anticipate a profitable and enjoyable session and it is hoped that representatives from evey local society in the United States wil be present.

JAS. McMANUS,
*Cor. Sec. American Dental Association.*

*Hartford, Conn.*

* This communication was received too late for its proper place under Correspondence.

